# Psychological, functional and social outcomes in adolescent and young adult cancer survivors over time: A systematic review of longitudinal studies

**DOI:** 10.1002/pon.5987

**Published:** 2022-07-02

**Authors:** Natalie K. Bradford, Fiona E. J. McDonald, Helen Bibby, Cindy Kok, Pandora Patterson

**Affiliations:** ^1^ Cancer and Palliative Care Outcomes Centre at Centre for Children's Health Research Queensland University of Technology Brisbane Queensland Australia; ^2^ Research, Evaluation and Social Policy Canteen Australia Sydney New South Wales Australia; ^3^ Faculty of Medicine and Health University of Sydney Sydney New South Wales Australia

**Keywords:** adolescent, cancer, neoplasm, oncology, survivorship, systematic review, young adult

## Abstract

**Objective:**

Most adolescents and young adults (AYA) can expect to survive a cancer diagnosis and treatment, but all will be left with the potential of long‐term negative effects that can impact their ability to reach their full potential in life. Understanding aspects of psychological, functional, and social health and well‐being outcomes, is pivotal for optimising long‐term well‐being.

**Methods:**

We completed a systematic review of longitudinal studies reporting outcomes after anti‐cancer treatment for Adolescents and Young Adults diagnosed between the age of 12–29 years according to established systematic review processes. The protocol was registered with PROSPERO (ID: CRD 42020203116).

**Results:**

Thirteen reports from 10 studies met eligibility criteria representing 17,645 individuals (50.3% female, mean age at diagnosis 22 years, and 26 years at last, follow up). Eleven reports were from eight quantitative studies that relied on self‐report surveys and two were qualitative studies. Psychological outcomes were reported to improve over time, as were functional health outcomes, although reported health behaviours were inconsistent between studies. Neurocognitive deficits were reported to affect the ability to return to work and impacts on fertility and sexuality were sustained over time.

**Conclusions:**

While some outcomes for AYA are reported to improve over time, particularly for physical functioning, and anxiety and depression, the long‐term impact of cancer on many important domains remains largely unknown. Specifically, the evidence to understand *what* changes occur over time, and *when,* remains underdeveloped.

## Key points

- Adolescents and young adults have a long time to live as survivors of cancer, and the negative effects of disease and treatment can compromise long‐term well‐being
- Longitudinal research is important for understanding changes in outcomes over time
- While a wide range of outcomes have been studied, the evidence to understand what changes occur and when remains underdeveloped

## 1 BACKGROUND

Globally, around 400,000 adolescents and young adults (AYA) aged between 15 and 29 years are diagnosed with cancer each year.^1^ Disease‐free survival rates in AYA are high, particularly in developed nations where across all cancers 83%–89% can expect to survive.^2^, ^3^, ^4^ The period of adolescence and young adulthood is, however, a crucial time for developmental changes psychologically, socially and physiologically, with an expected transition towards independence.^5^, ^6^ A cancer diagnosis at this time interrupts normal development and personal growth, and, coupled with the often intense and highly toxic anti‐cancer treatment, negatively affects multiple aspects of psychological, functional, and social wellbeing.

These negative effects do not abate with the end of treatment, which is recognized as a particular time of vulnerability when survivors experience heightened anxiety, stress, fear, and uncertainty about the future, combined with a perceived lack of support from healthcare services.^7^, ^8^, ^9^ While some young people find meaning in their experiences and report positive changes to their lives including post‐traumatic growth, for others, the negative effects of cancer and treatment can persist and may even worsen over time.^10^, ^11^ When negative impacts continue to affect psychological, functional and social aspects of life, a young person's identity, relationships, education, work, and finances are affected. Ultimately this may cascade into poor behavioural and emotional adjustment, as well as inhibiting achievement of developmental outcomes.^8^, ^12^, ^13^

Given that young people have a lifetime to live as survivors of cancer, they have enormous potential to positively contribute to society. After‐cancer follow‐up care typically focuses on clinical aspects of health‐related to surveillance for cancer recurrence and treatment‐related effects, but the persistent effects of cancer on psychological, functional, and social aspects of life must be also addressed.^14^, ^15^, ^16^ To inform the development of appropriate interventions for young cancer survivors, a clear understanding of the domains of life that are impacted, and the magnitude of such impact is required. Understanding these phenomena over time offers the opportunity to identify vulnerable touchpoints. This can inform gaps in understanding the effect of the cancer treatment trajectory at different developmental stages.

Longitudinal research, which examines variables over multiple time points, can advance knowledge about the onset, peak, and culmination or continuation of problems.^17^ Particularly when three or more time‐points are used, longitudinal research can be a powerful way to detect a change in constructs of interest.^18^ Despite the potential insights gained, longitudinal research in young people is often constrained by resources and other inherent barriers associated with research in small heterogeneous populations.^19^ While there are large population based studies that have reported outcomes for AYA during or soon after cancer treatment,^20^, ^21^, ^22^ there is a need to understand the longer term impacts on AYA post cancer treatment. To our knowledge, no previous review has synthesized outcomes from longitudinal studies after cancer treatment related to psychological health, physical health and functioning, and social aspects of life in AYA cancer survivors, including reported barriers to such research. Understanding how these outcomes are affected, and when changes occur, are crucial to understanding how to optimize long‐term health and well‐being. We thus aimed to systematically identify and synthesize the evidence from longitudinal studies of adolescents and young adults treated for cancer, and address the following questions:

1. What psychological, functional, and social domains are reported in longitudinal studies of AYA cancer survivors?
2. What outcomes from these domains are reported in longitudinal studies of AYA cancer survivors and how do they change over time?
3. What barriers and facilitators are reported in undertaking longitudinal studies with AYA cancer survivors?
4. What are the reported recommendations for future longitudinal research with AYA cancer survivors?

## 2 METHOD

The guidelines for Preferred Reporting Items for Systematic Reviews and Meta‐Analysis were followed throughout the review process.^23^ A protocol for the review was registered with PROSPERO (ID: CRD42020203116). Ethics approval was not required for this secondary analysis of data reported in primary studies. Data collected in primary studies were approved by a Human Research Ethics Committee.

### 2.1 Search for eligible studies

A systematic search using keywords and MeSH terms was conducted across databases including MEDLINE, EMBASE, Psychinfo, Web of Science, and CINAHL from 2005 until August 2020. Hand‐searching reference lists of retrieved articles was undertaken to identify other relevant reports. The database search was updated in August 2021. The full search strategy is available in Supporting Information S1.

### 2.2 Selection process

Dual processes were adhered to throughout the selection process. Retrieved reports were imported into Endnote™ where duplicates were removed. Titles and abstracts were independently screened for eligibility by two reviewers in Covidence (www.covidence.org). The full texts of reports were independently read by two reviewers, appraising each for inclusion according to the eligibility criteria. The review team met regularly via videoconference to discuss the process and resolve any discrepancies regarding the inclusion or exclusion of reports. All included (and excluded) reports were discussed and agreed upon by the whole review team (see Box 1 for inclusion/exclusion criteria).

**Box 1. Eligibility criteria**

**Table:** 

Inclusion criteria	Exclusion criteria
Longitudinal reports of survivors aged 12–29 years at the time of any type of cancer diagnosis Any type of cancer treated with any type of anti‐cancer treatment Quantitative, qualitative, or mixed methods reports that included primary data consisting of either self‐reported or direct objective measures of outcome variables of interest Outcome variables describing psychological, physical functioning, or social aspects of well‐being Outcome variables collected at least two times, and over a minimum of 12 months following the completion of anti‐cancer treatment Reports written in English and published in peer‐reviewed journals from 2005 onwards No restriction on report setting (primary, secondary or tertiary care)	Longitudinal reports where outcomes of the age range of interest (12–29 years) were not able to be separated from younger or older age groups Literature reviews, interventional or experimental studies, cross‐sectional studies, unpublished thesis, abstracts and conference proceedings Outcome variables measured at diagnosis or during treatment and were not able to be separated from outcomes measured post anti‐cancer treatment Unable to separate outcome variables collected during anti‐cancer treatment from those collected post‐treatment

**Box 2 Outcomes domains of interest**

**Table:** 

**Psychological health**
Anxiety
Depression
Distress
Fear of recurrence
Impacts on quality of life
Coping with challenges of cancer survivorship
**Functional health**
Physical functioning and health related quality of life
Neurocognitive outcomes
Health behaviours (sleep, diet, physical activity, smoking, drugs and alcohol)
Prevention and early detection of second cancers
**Identity and spirituality**
Impacts of cancer on identity
Spiritual needs and supports
Existential questions of meaning or purpose
Gender identity
**Intimate relationships, sexuality and fertility**
Intimate relationships with partner/spouse
Sexual health
Fertility and family planning
**Family**
Family relationships, communication, coping
**Social support**
Friendships, peer connections, community and other supports
**Education, work and leisure**
School, college or university attendance and attainment of qualifications
Employment and vocational issues
Participation in leisure activities
**Practical and financial issues**
Housing
Financial
Insurance
Travel

### 2.3 Population of interest

The population of interest were survivors of any type of cancer, who were treated for cancer as an adolescent, (defined as 12–19 years) or in young adulthood (defined as 20–29 years).^24^ While the age definition of AYA varies around the world, we chose this age range to be as inclusive as possible, while retaining a focus on the ages recognized as critical in the normal development of identity and establishing independence.^6^, ^13^ Where studies reported the outcome of survivors outside this age range, they were included only if data were reported separately for survivors in our age range of interest.

### 2.4 Outcomes of interest

Outcomes of interest were informed by a consultation with a small group of six AYA cancer survivors, and a review of the literature reporting conceptual frameworks for AYA survivorship care.^25^, ^26^ Multiple concerns were identified that were broadly grouped into eight domains: 1) psychological health; 2) functional health; 3) identity and spirituality; 4) intimate relationships, sexuality and fertility; 5) family; 6) social support; 7) education, work and leisure; and 8) practical and financial issues. Within each domain, multiple outcomes were identified as important; efforts were made to identify relevant reports of each outcome (see Supporting Information S1). Outcome domains are presented in Box 2.

### 2.5 Data extraction

Data were extracted using a data extraction template modified to suit our purpose in Covidence by one reviewer. Fifteen percent of included articles had a second reviewer independently extract data. Data extraction was compared and discussed with all authors, and discrepancies were resolved before the data from the remaining articles were extracted. Extracted data included: author, country and year; aims of the study; study setting; theoretical background; sampling approach; participant characteristics; data collection methods; study duration and data collection time points; primary and secondary outcome domain category, mean differences in outcome variables; author explanations of the key findings; reported barriers and facilitators, calculation of attrition rates, and author recommendations for future research.

### 2.6 Quality appraisal

The Quality Assessment Tool for Studies with Diverse Designs (QATSDD) critical appraisal tool was used to appraise the quality of each study,^27^ again using Covidence. Two reviewers appraised and scored each article independently with disagreements resolved through discussion with a third reviewer. Each item of the QATSDD was scored from 0 = not at all, to 3 = complete, to give a total possible score of 42, with higher scores indicating higher quality.^27^ Scores were summed and then transformed into a percentage rating, again with a higher rating indicating higher quality. No studies were eliminated based upon quality rating; rather quality scores were used to appraise the completeness of reporting and to generate recommendations for future studies undertaking and reporting longitudinal research.

### 2.7 Strategy for synthesis

Participant characteristics from the pool of studies were tabulated including the number of studies, number of total participants, tumour type, participants' age and sex, and other relevant clinical/demographic information if available. Content analysis was used to synthesize extracted findings from various types of study designs.^28^ Coding categories at various levels were applied to the segments of systematically extracted data using a deductive approach. The sample, data, methods, and outcomes of each study were the focus of the synthesis, and data were organized according to pre‐determined structures. Matrices were used to organize data within and across studies.^29^ Results are presented in a narrative description and tables.

## 3 FINDINGS

**FIGURE 1:**
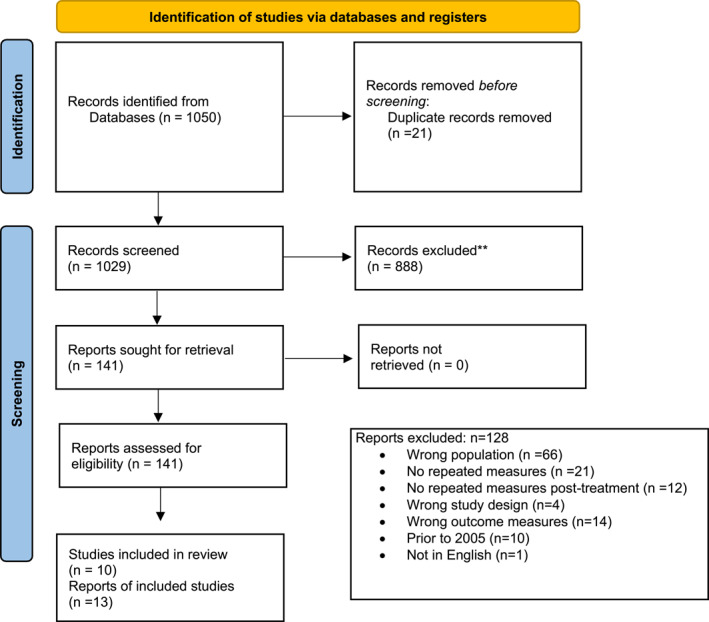
PRISMA flow diagram of study selection process

Of the 1050 articles screened, the full texts of 141 papers were reviewed resulting in 13 reports from 10 studies included in this review (Figure 1).^30^ Studies were undertaken between the years 2007–2021 and at baseline represented a total of 17,645 (50.3% female) AYA diagnosed with cancer in Europe (*n* = 7)^31^, ^32^, ^33^, ^34^, ^35^, ^36^, ^37^ and North America (*n* = 6).^38^, ^39^, ^40^, ^41^, ^42^, ^43^ Five studies did not report attrition, providing data only for participants that had contributed data at two or more time points.^31^, ^37^, ^40^, ^41^, ^43^ Across the remaining studies, 1325 AYA (7.5%) were lost to follow‐up and did not contribute at all time points, leaving a total of 16,320 participants contributing at all time points across all studies. The smallest study reported outcomes for 16 AYA,^31^ and the largest study recruited 9416 AYA.^42^

There were three reports^40^, ^41^, ^42^ that met our inclusion criteria from the broader St Jude Research Hospital's large Childhood Cancer Survivor Study (CCSS).^44^ Two reports were included from the AYA Leipzig study from Denmark.^36^, ^37^ Across all included reports the mean age at diagnosis was 21 years, and 26 years at last follow up. A wide variety of cancer diagnoses were represented. Studies ranged in duration from 12 months to up to 13 years, with data collected a mean of 2.7 times, and up to five times in the larger studies (see Supporting Information S2). Seven studies were quantitative longitudinal designs with no comparator group.^32^, ^35^, ^36^, ^37^, ^38^, ^39^, ^43^ Four studies were quantitative longitudinal cohort studies with a comparative control group,^33^, ^40^, ^41^, ^42^ and two were qualitative longitudinal studies.^31^, ^34^ Characteristics for studies are summarised in Supporting Information S2, and in Table 1 a matrix of primary and secondary outcome domains reported in each study is tabulated.

**TABLE 1:** Reported outcome domains

Study reference (number of participants)	Psychological health	Functional health			Identity and spirituality	Intimate relationships, sexuality and fertility	Social	Education, work and leisure	Practical and financial issues
		Health behaviours	Physical functioning and HRQoL	Neurocognitive					
Acquati 2018 (123 at baseline)	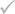		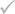			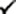	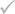		
Armuand 2018 (16 at baseline)						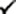			
Bekkering 2012 (41 at baseline)			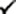						
Brinkman 2019 (4484 at baseline)	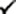	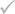		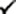					
Brock 2021 (502 at baseline)				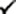				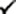	
Capelli 2021 (127 at baseline)		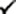							
Cho 2015 (120 at baseline)		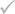	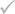		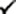				
Daniel 2019 (2645 at baseline)	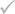	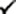	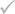						
Gibson 2015 (4997 at baseline)		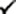							
Gregurek 2009 (109 at baseline)	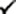		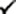			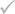		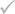	
Jorngarden 2007 (56 at baseline)	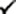		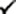						
Lehmann 2014 (28 at baseline)	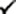		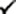		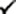	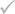	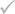		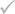
Leuteritz 2018 (577 at baseline)	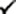		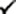		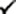		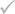		

*Note*: 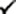, primary outcome; 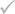, secondary outcome.

Abbreviations: HRQoL, Health related Quality of life.

### 3.1 Quality appraisal

The quality appraisal of individual reports ranged from 38% to 87% (mean 66%) in terms of percent total score across all items. Reports generally provided a clear statement of their aims and objectives and a rationale for the choice of data collection tools. No reports had evidence of the participant, public, or AYA involvement in the study design or interpretation of findings. Generally, poorly reported items included the statistical assessment of the reliability and validity of measurement tools and evidence of consideration of the sample size in statistical analysis. Details of the quality appraisal for each study are presented in Supporting Information S3.

### 3.2 Reported domains and outcomes

The outcome domains available from each report are summarised in Table 1 and described below. Most included reports described multiple outcome domains; secondary outcomes are tabulated but not further described.

#### 3.2.1 Psychological health outcomes

Anxiety and depression were the focus of two studies (158 baseline participants).^33^, ^35^ Both these studies reported decreases in state and trait anxiety over time, which was positively correlated with improved quality of life. While baseline measurement indicated depression and anxiety were overrepresented in young people with cancer, the reported improvements suggest that young people adjust over time (within 18 months).^33^, ^35^ A smaller qualitative study with just 28 baseline participants hypothesized psychological issues, along with physical health, were most likely to be reported as negative consequences of a cancer diagnosis during adolescence; findings however suggest both positive and negative consequences of cancer diagnosis occur that change with time.^34^

#### 3.2.2 Functional health outcomes

Within the functional health domain, health behaviours (sleep, smoking, alcohol use) were the focus of three studies (7769 baseline participants).^41^, ^42^, ^43^ Capelli et al. (2021) found binge drinking and smoking both tobacco and marijuana were more prevalent in young cancer survivors compared to population norms but acknowledged more research is required to clarify these findings. Conversely, the Gibson et al. study (2015) found smoking rates decreased over time, and that development of adverse health conditions was not associated with smoking.^42^ Daniel et al. (2019) identified poor sleep behaviours were associated with late‐onset or persistent psychological distress, and that insomnia, fatigue, and use of medication for sleep were associated with negative physical conditions including hypertension and headaches.^41^

Only one included study focussed specifically on physical function, using body‐worn accelerometer sensors to objectively measure physical activity after surgical interventions for malignant tumours (40 baseline participants).^32^ This study identified that young cancer survivors improved in their functional and physical activity over the study duration (24 months), with the most pronounced improvement made in the first 12 months. Four other studies measured, or enquired about, physical functioning as an aspect of health‐related quality of life but did not report specifically on physical functioning.^33^, ^34^, ^35^, ^36^

The impact of drug and alcohol use on neurocognitive function (4484 baseline participants) was the focus of one large childhood cancer survivorship self‐report study. Risky drinking, including drinking before age of 18 years, was associated with memory problems and risks of persistent psychological distress.^40^ Brock et.al (2021) was specifically interested in the effects of cognitive impairment upon return to work (502 participants at baseline) and identified workability improved over time.

#### 3.2.3 Identity and spirituality outcomes

Three studies examined constructs categorised in the domain of identity and spirituality including ‘cancer‐related identity’ (120 baseline participants),^39^ positive and negative effects of cancer on life (61 baseline participants),^34^ and life satisfaction (577 baseline participants).^36^ All three studies concluded further research was required to better understand the potential interplay of coping strategies, perceived severity, identity, empowerment, and self‐efficacy. Cho and Park (2015) identified the importance of language when referring to or describing young people affected by cancer.^39^ No studies included a focus on spirituality.

##### Intimate relationships, sexuality, and fertility outcomes

Two included studies (144 baseline participants) reported on aspects of relationships, sexuality, or fertility.^31^, ^38^ The study by Acquati et al. found that 52%–54% of young cancer survivors reported problems with sexual function, sustained at 24 months; this was accompanied by increases in psychological distress over time.^38^ Armuand et al. (2018) undertook a qualitative study using semi‐structured interviews over 24 months with 16 participants and explored how AYA cancer survivors experienced infertility treatment over time and concluded that fertility‐related communication should be included as standard care in the cancer treatment of young people.^31^

#### 3.2.4 Education, work, and leisure outcomes

One study (baseline 502 participants) examined workability and associated cognitive impairment resulting from cancer treatment in young people.^37^ While mean workability significantly increased over time, 57% of participants reported persistently decreased workability after 12 months; cognitive impairments and other comorbidities were significantly associated with decreased workability.^37^

#### 3.2.5 Outcome domains of interest not included

This review sought to explicitly identify longitudinal articles reporting on other domains aside from those reported above including family relationships and communication, social support including connection with peers and community, education and leisure activities, practical and financial issues, and spirituality. While some studies did explore some of these concepts as secondary outcome measures, there were limited data available for synthesis. These domains remain largely unstudied in the post treatment phase of survivorship for AYA with cancer and thus impacts these aspects of life are not well understood.

### 3.3 Reported barriers and facilitators to longitudinal research in AYA cancer

Challenges with recruitment, resulting in small sample sizes were reported in several studies.^31^, ^33^, ^34^, ^39^, ^43^ High attrition rates and challenges to follow up with participants were also reported.^32^, ^38^ We calculated attrition rates ranging between 11% and 63% for the studies that reported participant numbers at different time points. For some of the larger studies, for example, the CCSS studies, outcome data were only presented for the participants who had provided data at more than one‐time point and attrition rates were not reported, nor able to be calculated. Other studies acknowledged that potential selection bias may have affected the reported findings.^37^ For example, in the AYA‐Leipzig study, while 577 participants were recruited at baseline, 75% were female and the reason for refusal and refusal rates were not systematically documented^36^ In the larger CCSS studies, recall bias is acknowledged as a potential confounding factor, particularly for reporting health behaviours such as sleep, smoking or alcohol consumption.^40^, ^41^, ^42^ Despite the clear need for longitudinal research in this population group, there were no specific facilitators reported to undertake such research. Of note, 10 of the 13 studies included in this review were reports of a larger study (e.g. CCSS studies) or a report of a component of another published study suggesting researchers in this area rely on mining existing datasets to address research questions. Some of these studies reported on data collected decades earlier, for example, one study reporting effects of sleep, emotional distress and physical health reported on data collected between 1994 and 2007.^41^

### 3.4 Recommendations for future research

Among the included reports, it was widely recognised that more longitudinal studies with larger sample sizes were required to understand the impacts and severity on long‐term health and well‐being of AYA cancer survivors.^33^, ^34^, ^39^, ^40^, ^41^, ^43^ The need to actively promote retention was less widely acknowledged.^38^

Some studies recommended more frequent assessments, using measures validated for the specific age group,^37^, ^39^ and also the importance of developing measures for specific domains such as cancer‐related identity.^39^ Including a control group to enhance rigour was also recommended to help understand and identify targets for interventions.^32^, ^37^, ^38^, ^43^

Other recommendations included exploring the role of coping, self‐efficacy and empowerment^36^; examining the role of employer support or other occupational/vocational related factors that may have an impact on the ability to return to work or education^37^; investigating the role of specific medications and their duration of use to understand how these impact health and social functioning^41^; and developing interventions to mitigate persistent negative health behaviours.^42^

## 4 DISCUSSION

To our knowledge, this is the first review to systematically identify, appraise, and synthesise the available longitudinal studies post cancer treatment in AYA diagnosed with cancer. Despite a wide search across multiple databases, only 13 reports from 10 studies met eligibility criteria and were included in this review. Across these studies, we have synthesized findings on outcomes including psychological health, functional health and behaviours, identity, sexuality and fertility, and work. We also report the documented barriers to undertaking longitudinal research and recommendations for future research. Studies were heterogeneous, and reports varied in the quality of information presented, such that identifying systemic barriers or facilitators to longitudinal research remains elusive. This is not a new problem, with previous studies also identifying challenges to recruitment and retention of AYA with cancer.^45^

Previous reviews of studies involving AYA cancer survivors have explored concepts such as unmet needs,^46^ social well‐being,^16^ as well multiple outcomes reported in 17 papers associated with the AYA HOPE study.^20^ Additionally a recent scoping review maps the purpose of 161 different AYA cancer studies.^47^ While it is encouraging to see the breadth of topics and domains synthesised in such reviews, the evidence to understand *what* changes occur over time, and *when*, remains underdeveloped and unknown. Although some studies included in our review identified improvements in outcomes over time, particularly for physical functioning,^32^ and anxiety and depression,^33^, ^35^ other studies identified little improvement in outcomes over time for a significant proportion of AYA.^37^, ^38^, ^40^, ^41^ Moreover, while there are a few longitudinal studies that encompass issues pivotal to the development of young people, there were no longitudinal studies that focussed on several of the domains we had identified as being important to young cancer survivors, including family relationships, social support, spirituality, education, leisure and practical and financial issues.

We identified that the attrition rates reported in the included studies could be potentially misinterpreted as the eligibility criteria were re‐defined in some studies after baseline enrolment. Some studies included longitudinal data for participants at different time points which were then combined for analysis reporting retention at only one point in time. This type of reporting may deflate the actual attrition rate and provide little information for understanding the complexities of undertaking longitudinal research. Moreover, high attrition leads to a substantial bias in results, particularly if the missing data is non‐random.^48^ It is important to determine and report the cause of attrition, as any significant differences in characteristics of participants at different time points may be indicative of systematic non‐response bias.^18^

Of note regarding the quality appraisal, we identified no studies that reported the inclusion of patient and public involvement in either research design or interpretation of outcomes. While not explicitly stated in included studies, many long‐standing studies do have established patient advisory boards. A recent systematic review identified that the inclusion of people with the lived experience of the condition being studied was significantly associated with improved enrolment in studies, and although inconclusive regarding retention, this warrants consideration.^49^ The inclusion of young people with cancer in the design of studies helps ensure outcomes of importance are included and may reduce attrition.^50^ It is also important to report inclusion of stakeholders and consumers in dissemination.

The concept of time and the domains measured in quantitative longitudinal research offers aggregated metrics to represent change and provide potential explanatory reasons to understand the predictors and causes of change.^18^ However, before attempting to explain what has caused a change, and when this may have occurred, a theoretical understanding of the reason for the change is required. To form such a theory, a description of the change overtime is required. This kind of understanding may be better characterized in qualitative longitudinal studies, which are valuable for exploring complex phenomena over time and developing a deeper understanding.^51^ Indeed, qualitative longitudinal research is recognized as a powerful tool for identifying critical moments and broad patterns in experiences that then may inform interventions.^52^ This review identified only two longitudinal qualitative studies.^31^, ^34^ Practical, financial, and resource constraints limit undertaking studies that can truly understand phenomena, predict their cause and develop, implement and evaluate an intervention to address identified problems.^18^ This is an evident barrier demonstrated by the numerous reports included in this review that report sub‐studies of a larger program of research.

### 4.1 Study limitations

This review was undertaken according to the established methodologies for systematic reviews, including dual processes for screening, appraising, and extracting data. While we had clear eligibility criteria, including only young people who were diagnosed as an AYA, this may have inadvertently resulted in exclusion in potentially relevant studies. While someone diagnosed when a younger child will still transition through adolescence and adulthood, their cancer experience is different and hence its impact on them will also be different.^14^ Likewise, requiring the data to have been collected at least twice during survivorship, meant that changes observed were associated with the survivorship phase while an AYA. These criteria did exclude several papers where baseline data had been collected during treatment, and where it was unclear whether the AYA had two clear data points after treatment ended over a minimum of 12 months. There was attrition over time in most reports that may have impacted their findings.^53^ A further limitation of this review is that we included only studies published in English, and all studies were from either the USA or Europe. While our search was broad, we may have missed studies. There remains a paucity of evidence to truly understand the long‐term outcomes for AYA after cancer diagnoses and treatment.

### 4.2 Clinical implications

The upheaval of a cancer diagnosis as a young person can disrupt optimal development with lifelong implications. Understanding these effects over time enables appropriate services and support to be developed and funded. Further longitudinal research is required to identify predictors of outcomes and target at‐risk young people and tailor service provision. Research is also required to better understand the interplay of coping strategies, perceived severity, identity, empowerment, and self‐efficacy, family relationships, social, practical and financial supports and communication. Outcomes from longitudinal research are critical to understanding change over time, and for informing potential interventions.^37^, ^40^, ^42^ While such research should be prioritized, there are inherent theoretical, methodological, and design considerations that require careful thought. The timing and number of repeated measures must be carefully considered if planning to make inferences from analyses; including at least three‐time points add rigour to the ability to detect change.^18^, ^51^ Planning for attrition is important, particularly in a young population that are more likely to change address or lose interest in contributing to a study.^54^ Active strategies are required to promote retention.^38^ Longitudinal qualitative studies may offer important insights about how and why change occurs that are not able to be measured quantitatively. Where quantitative research is undertaken, it is important to ensure measures are validated for the age group, sensitive to change over time, relevant and appropriate—yet comprehensive and multidimensional. Attention to these factors would enhance the understanding and meaning of outcomes.^32^, ^33^, ^37^, ^41^ Additionally, including an age‐matched control group would enhance the rigour of findings.^32^, ^33^, ^37^, ^38^, ^43^

The lack of psychometrically sophisticated measures for the AYA population with cancer is a highlighted research gap.^39^, ^55^ Domains such as coping, identity, sexual functioning, self‐efficacy, empowerment, and perceived severity of cancer consequences are areas that require further development.^34^, ^38^, ^39^ Future research should carefully consider the challenges of research in this area and look to include a broader range of outcomes important to young people.

## 5 CONCLUSIONS

Longitudinal studies of AYA with cancer are important for understanding how outcomes change over time. This review identified a wide range of outcomes important to AYA cancer survivors that have been studied longitudinally in either the USA or Europe. Although some studies identified improvements in outcomes over time, particularly for physical functioning, and anxiety and depression, other studies identified little improvement in outcomes over time for a significant proportion of AYA, indicating a critical need for greater understanding and intervention. The evidence to understand *what* changes occur over time, and *when,* remains underdeveloped.

## AUTHOR CONTRIBUTIONS

Pandora Patterson, Fiona McDonald and Helen Bibby conceptualized the study. All authors contributed to the design and execution of the methodology. Analyses were undertaken by Cindy Kok, Helen Bibby, Natalie K. Bradford and Fiona McDonald. Drafting of the manuscript was undertaken by Natalie K. Bradford. Review and editing were completed by all authors.

## CONFLICT OF INTEREST

The authors declare no conflicts of interest.

## ETHICS STATEMENT

This review did not involve human participants or animal subjects. This review is a secondary analysis of data obtained from peer‐reviewed primary studies and did not require specific ethics approval.

## Supporting information

Supporting Information S1

Supporting Information S2

Supporting Information S3

## Data Availability

The data that support the findings of this study are available in electronic databases. These data were derived from the following resources available in the public domains: MEDLINE, EMBASE, Psychinfo, Web of Science, and CINAHL.
