# Supplementary material for: Psychological, functional and social outcomes in adolescent and young adult cancer survivors over time: A systematic review of longitudinal studies
Source: Psychooncology. 2022 Jul 2;31(9):1448–58. doi: 10.1002/pon.5987 (PMC9544373; doi:10.1002/pon.5987)
Supplement: Supplementary file 1 — Supporting Information S1 [file PON-31-1448-s003.docx]

| **Population of interest** | |
| --- | --- |
| #1 | "cancer survivors"[MeSH Terms] OR cancer survivor[Text Word] OR **"Cancer Survivors" OR "Long-Term Cancer Survivors" OR "Survivors of Childhood Cancer")**"cancer survivors"[MeSH Terms] OR cancer survivor[Text Word] OR **"Cancer Survivors" OR "Long-Term Cancer Survivors" OR "Survivors of Childhood Cancer")** |
| #2 | **"Adolescence" OR "Adolescent"[MeSH Terms] OR "Adolescent" OR "Adolescents" OR "Adolescents, Female" OR "Adolescents, Male" OR "Teenagers" OR "Teens" OR "Youth OR** "young adult"[MeSH Terms] OR young adult[Text Word] OR "child"[MeSH Terms] OR child[Text Word] OR"p*ediatrics"[MeSH Terms] OR p*ediatric[Text Word] |
| **Study design** | |
| #3 | **"Longitudinal Studies"[MeSH Terms] OR "Longitudinal Studies" OR "Longitudinal Survey"** |
| **Outcomes** | |
| #4 | “psychological distress” OR “psychological adjustment” OR “psychological adaptation” OR “depression” OR “anxiety” OR “emotional distress” OR “emotional adjustment” OR “emotional adaptation” OR “distress” OR “stress” OR “quality of life” |
| #5 | "patient outcome assessment"[MeSH Terms] OR (("patient"[All Fields] AND "outcome"[All Fields]) AND "assessment"[All Fields])) OR "patient outcome assessment"[All Fields] |
| #6 | "fertility"[MeSH Terms] OR "fertility"[All Fields] |
| #7 | behavior” [MeSH] OR “ behavior”[All Fields] OR “Learning Disabilities” [MeSH] OR “Learning Disabilities” [All Fields] |
| #8 | ("intimate"[All Fields] OR "intimates"[All Fields]) AND ("relationship"[All Fields] OR "relationships"[All Fields]) OR“friends”[MeSH terms] OR ‘friends’[All Fields] OR“peer group”[MeSH terms] or “peer*[All Fields] OR “interpersonal relations”[MeSH terms] or “interpersonal relations” [All Fields”] OR ((("family relations"[MeSH Terms] OR ("family"[All Fields] AND "relations"[All Fields])) OR "family relations"[All Fields]) OR ("family"[All Fields] AND "relationship"[All Fields])) OR "family relationship"[All Fields] OR ("social"[All Fields] OR "socialisation"[All Fields]) OR "socialization"[MeSH Terms]) AND ("relationship"[All Fields] OR "relationships"[All Fields]) |
| #9 | "education"[MeSH Terms]) OR "education s"[All Fields]) OR "employability"[All Fields] OR "employable"[All Fields] OR "employer"[All Fields] OR "employer s"[All Fields] OR "employers"[All Fields] OR "employment"[MeSH Terms] OR "employment"[All Fields] OR "employments"[All Fields] OR "work"[MeSH Terms] OR "work"[All Fields] OR  (((("leisure activities"[MeSH Terms] OR ("leisure"[All Fields] AND "activities"[All Fields])) OR "leisure activities"[All Fields]) OR "leisure"[All Fields]) OR "leisures"[All Fields]) OR "leisurely"[All Fields] OR "economics"[MeSH Subheading] OR "economics"[All Fields] OR "finances"[All Fields] OR "economics"[MeSH Terms] |
| #10 | "identities"[All Fields] OR "identity"[All Fields] OR "spirituality"[MeSH Terms] OR "spirituality"[All Fields] OR “faith”[All Fields] OR ‘religion”[All Fields] OR “existential” [MeSH Terms] OR "existential"[All Fields] OR "sexuality"[MeSH Terms] OR "sexuality"[All Fields] |
| #11 | #1 AND #2 |
| #12 | #11 AND #3 |
| #13 | #4 OR #5 OR #6 OR #7 OR #8 OR #9 OR #10 |
| #14 | #12 AND #13 |
