# Supplementary material for: Psychological, functional and social outcomes in adolescent and young adult cancer survivors over time: A systematic review of longitudinal studies
Source: Psychooncology. 2022 Jul 2;31(9):1448–58. doi: 10.1002/pon.5987 (PMC9544373; doi:10.1002/pon.5987)
Supplement: Supplementary file 2 — Supporting Information S2 [file PON-31-1448-s001.docx]

| **Reference (study name if applicable)**  **Country/ Range of years data collected** | **Aim and Study design**  **Methods for data collection (include comparison group)**  **Duration followed,**  **Time-points and intervals** | **N= sample**  **(attrition %)**  **Study population characteristics** | **Primary outcome category/ Domain(s) (tool)**  **Secondary outcome category/ Domain(s) (tool)** | **Summary of outcomes (including significant predictors)** | **Reported barriers and limitations** | **Recommendations for further research** |
| --- | --- | --- | --- | --- | --- | --- |
| Acquati (2018)  Country  USA  Data collected 2008-2012 | Aim:  To examine the prevalence of sexual dysfunction  Study design: Longitudinal quantitative, multisite  Methods: AYA survivors recruited from hospital clinics completed surveys  Duration followed:  24 months  Time points and intervals  3 time points  T0 (within 4 months from diagnosis),  T1=6 months later  T2=24 months later | Number recruited:  T0 Baseline= 123,  T1= 107,  T2= 95  (attrition = 22.76%)  Sex: Male =53.7%  Diagnosis: Multiple cancers  Age at diagnosis:  mean 28.2 years (range 18-39 years)  Age at recruitment:  Within 4 months of diagnosis | Primary Outcome category:  Intimate relationships, sexuality & fertility  Domains measured (tool) Sexual functioning (MOS Sexual Functioning Scale total score)  Secondary Outcome categories:  Psychological health; Functional health Social  Domains measured (tool) Psychological distress (GSI-BSI-18); HRQoL (SF-36); Social support (MOS Social Support Survey) | A substantial proportion (52-54.2%) report some degree of problem with sexual functioning, which was sustained at 24 months.  Psychological distress increased over time.  Physical and mental components of HRQoL improved over time but were still below the population mean.  Social support remained elevated over time.  Worse sexual function was predicted for females, older, being in a relationship, receiving chemo, higher distress, lower social support. | Barriers:  Attrition rate highlights challenges of recruiting and following up AYA.  Limitations:  The more frequent assessment would provide more accurate estimates of sexual dysfunction | Studies that actively promote retention are required.  The study could be enhanced by using a multi-dimensional measure of sexual functioning, conducting more frequent assessments, and including a matched control group. |
| Armuand (2018)  Country  Sweden  Data collected  2009-2011 | Aim:  To explore how men and women experience the threat of infertility and their thoughts about having children after cancer during the first 2 years following diagnosis  Study design:  Longitudinal qualitative study  Data collection methods:  Recruitment from 3 different oncology and haematology wards at a university hospital  Duration followed:  24 months  Time points and intervals  2-time points  T0= Baseline  T1= 2 years later | Number recruited:  T0 =16  T1=16  Sex:  Male =43.8%  Diagnosis:  Multiple cancers  Age at diagnosis:  Not reported  Age at recruitment: mean 32.0 years (range 23-41) | Primary Outcome category:  Intimate relationships, sexuality & fertility  Domains measured (tool)  Thoughts and feelings about threatened infertility and having children (interview)  Secondary Outcome categories:  Nil | Four themes were reported: Continue calmly on the chosen path, Abandoning plans for children, Avoiding the subject of fertility, and Struggling towards life goals.  Recommendations to offer individualised fertility-related treatment communication and counselling, both at the time of cancer diagnosis and also in connection with follow-up care. | Barriers:  Not reported  Limitations:  Relatively small sample group, 24 months between data collection time. | No recommendation for future research |
| Bekkering (2012)  Country  Netherlands  Data collected  2004-2008 | Aim:  To assess QoL, functional ability, and physical activity levels in children and adolescents during the first 2 years after surgical intervention for a malignant tumour around the knee joint  Study design: longitudinal assessments  Data collection methods: Recruited from 3 university medical centers  Duration followed:  24 months  Time points and intervals  5 time points  T0=3 mths post-surgery  T1= 6 mths post-surgery  T2=9 mths post-surgery  T3=12 mths post-surgery  T4=18 mths post-surgery  T5=24 mths post-surgery | Number recruited:  T0 =41  T5=24  (attrition=42%)  Sex:  Male =61%  Diagnosis: Osteosarcoma, Ewing sarcoma  Age at diagnosis:  mean 14.9 years (SD4.8)  Age at recruitment:  not reported | Primary Outcome category:  Functional health  Domains measured (tool)  HRQoL (Under 16: TACQOL. 16+: TAAQOL. All: SF-36, BT-DUX); Functional ability (TESS, TUDS time, VWA time, 6 MWT distance & PCI); Physical activity (Baecke questionnaire, ActiLog GPA & number of high activity periods per day)  Secondary Outcome categories:  Nil | Over the first year, survivors showed significant improvement of QoL, functional ability, and physical activity, except for the mental dimension of the SF-36 and the activity monitor results.  Over the second year, these improvements were less pronounced. | Barriers:  Not reported  Limitations:  in the early postoperative period patients have to adapt to the new limb thus the localization of the tumour may have played a role  All patients received postoperative chemotherapy, which is likely to have had a significant impact on their QoL and functional ability | Future research should be built on some of the strengths of this study such as the prospective, longitudinal design. Future research should include patients from various age groups; and a comprehensive set of validated measures of QoL, functional ability, and physical activity. |
| Brinkman (2019)  (Childhood Cancer Survivor Study)  Country  North America and Canada  Data collected  1970-1986 (unable to determine exact time-points for this study) | Aim:  (1) estimate the level of alcohol consumption behaviours, (2) compare alcohol consumption behaviours between survivors and sibling controls, (3) test associations between alcohol consumption behaviours and (a) neurocognitive impairment and (b) symptoms of emotional distress  Study design: a retrospective cohort study with longitudinal follow up  Data collection methods: Survivors of childhood cancer recruited from 26 institutions across North America and Canada  Duration followed:  48 months  Timepoints and intervals  3 timepoints  T0= Baseline  T1=24 months  T2= 48 months | Number recruited: n=4484  (attrition N/A)  Sex: Male = 52.7%  Diagnosis: Multiple cancers  Age at diagnosis: mean 10.5 (SD 5.6)  Age at recruitment:  mean 27.2 years (SD 6.2) | Primary Outcome categories:  Functional health; Psychological health  Domains measured (tool)  Neurocognitive functioning (CCSS-NCQ); Emotional distress (BSI-18: depression, anxiety and somatization subscales, PDS)  Secondary Outcome category:  Functional health  Domains measured (tool)  Alcohol consumption (risky drinking, heavy drinking, persistence heavy/risky drinking, age at drinking initiation) | After  adjustment for childhood cancer treatment exposures, including cranial radiation therapy (CRT), drinking initiation before  18 years of age was associated with a 30% increased risk of subsequent memory problems, depression, anxiety, and somatization.  Persistent  heavy/risky drinking was associated with an 80% increased risk of persistent psychological distress.  Compared with siblings, survivors were significantly less  likely to report heavy drinking, risky drinking, persistent heavy/risky drinking  and consuming their first drink before 18 years of age. | Barriers:  Not reported  Limitations:  The study relied on self-report of neurocognitive problems  Recall bias may influence self-report of alcohol consumption behaviours | Additional prospective longitudinal studies are necessary  to further elucidate the associations between alcohol consumption and emotional distress in survivors of  childhood cancer.  Because survivors are at increased  risk of developing chronic health conditions, future  efforts should aim to more clearly understand the  impact of these highly prevalent health morbidities on  neurocognitive functioning |
| Brock (2021)  (AYA-Leipzig study)  Country  Germany  Data collected  2014-2016 | Aim:  To investigate the extent of workability and cancer-related cognitive impairments in AYA cancer survivors and whether both changed over time and its relation to sociodemographic, medical, and psychosocial variables  Study design: a longitudinal study  Data collection methods: recruited from acute oncological clinics  Duration followed:  12 months  Timepoints and intervals  2 timepoints  T0= Baseline  T1= 12 months | Number recruited:  T0 =502  T1 =502  (Attrition N/A)  Sex:  Male = 25.3%  Diagnosis:  Multiple cancers  Age at diagnosis: mean 29.7 years  (SD 6.1)  Age at recruitment:  mean time since diagnosis 12.15 years (SD 8.21) | Primary Outcome categories:  Education, work & leisure; Functional health  Domains measured (tool)  Workability (single items from the WAI); Cognitive impairment (4-item cognitive impairments subscales from the COPSOQ)  Secondary Outcome categories:  Nil | Mean workability increased significantly over time.  76% of AYA cancer survivors reported reduced workability at baseline; 1 year later, this still applied to 57% of them.  Decreased workability at follow-up was associated with more cognitive impairment, higher effort coping with the disease, comorbidities, sick leave > 6 months (since diagnosis), and having children.  Cognitive impairments occurred in 18% of patients at baseline and 16% at follow-up. | Barriers:  Not reported  Limitations:  women and a high level of education were overrepresented in the study.  The option for patients to register themselves for the study might have resulted in selection bias.  The COPSOQ has not been validated in cancer populations | The COPSOQ should be validated with cancer patients and survivors.  Future research should explore the role of other factors, such  as employer support or additional work-related and psychosocial  factors, that might have a long-term negative impact on  the workability of AYAs and examine what support AYAs need to successfully re-enter education or employment. Comparisons with healthy peers would also be helpful. |
| Capelli (2021)  (Project Forward)  Country  USA  Data collection  2007-2015 | Aim:  To examine associations between risk and protective factors for substance use among a sample of Young Adult Childhood Cancer Survivors  Study design: a longitudinal study  Data collection methods: patients who had been diagnosed with any cancer (except Hodgkin lymphoma) were recruited from two large paediatric medical centres  Duration followed:  Minimum 5 years  Timepoints and intervals  2 timepoints  T0 between 2007-2009 T1 on average 5.1 years later. | Number recruited:  T0=127  T1=127  (Attrition N/A)  Sex: Male = 45%  Diagnosis: Multiple cancers  Age at diagnosis: mean 12.4 years (SD 2.4)  Age at recruitment:  mean 20.9 years (SD 2.8) | Primary Outcome category:  Functional health  Domains measured (tool)  Substance use (cigarette, alcohol and marijuana use in the past 30 days, binge drinking (5+ drinks on one occasion) in the past 30 days)  Secondary Outcome categories:  Nil |  | Barriers:  Not reported  Limitations:  small sample size, not compared to non-cancer peers, the survey did not include preventative measures | Future longitudinal work with larger samples is needed.  Future work should include  an age-matched control group to further investigate protective  and risk factors of drug use in comparison with  cancer non-affected peers.  Future research should measure the type and quantity of substance use counselling received to clarify the relationship with future substance use to more finely target prevention efforts. |
| Cho (2015)  “Part of a larger study of cancer survivorship and  quality of life in late adolescents and young adults” – name not stated.  Country  USA  Data collection  Years not stated | Aim:  Examined several questions regarding cancer-related identity in AYAs diagnosed with cancer:  Will participants more often endorse the terms ‘survivor’ and ‘person who has had cancer’ than the terms ‘patient’ and ‘victim’.  Are cancer-related identities are associated with participants’ adjustment?  Are participants higher on survivor or conqueror identities will report higher HRQOL and more positive impact.  Do some identities that might empower relate to better adjustment?  Study design:  longitudinal study  Data collection methods: Young adult cancer survivors identified through a regional hospital cancer registry  Duration followed:  12 months  Timepoints and interval  2 timepoints  T0=Baseline  T1 = 12months | Number recruited:  T0 =120  T1 =84  (attrition = 30%)  Sex:  Male = 21%  Diagnosis:  Multiple cancers  Age at diagnosis: mean 28.95 years (SD 6.82)  Age at recruitment: mean 32.59 years (SD 7.43) | Primary Outcome category:  Identity & spirituality  Domains measured (tool)  Cancer-related identity (researcher-created scale)  Secondary Outcome categories:  Functional health  Domains measured (tool)  Positive and negative impacts of cancer (IOC); HRQoL (Quality of Life Index); Health behaviours (researcher-created scale) | AYA with cancer identify with multiple terms. “Someone who has had cancer” was the most endorsed identity at baseline and follow-up.  “Survivor” and “member of the cancer community” were also frequently chosen.  Endorsement of “patient” and “victim” identities decreased over time.  Baseline demographics and clinical variables were associated with adjustment at follow-up.  Treatment type was associated with cancer identity, the impact of cancer, and HRQoL at follow-up.  No associations were found between cancer identity and health behaviours. | Barriers:  Not reported  Limitations:  A preliminary and exploratory study,  majority of participants were female and Caucasian  Because of multiple comparisons, results are vulnerable to inflated Type I error | More psychometrically sophisticated measures of cancer identity are needed.  More studies are needed to examine changes in cancer-related identities across  time, and to look further into the “member of the cancer community” identity.  The study should be replicated with a larger sample, longer assessment period, and more measurement points.  Future studies should investigate predictors of ‘empowering’ identities such as “survivor”, investigate whether people can incorporate these terms in their lives and live by these terms, and explore the processes by which cancer-related identities lead to adjustment. |
| Daniel (2019)  (Childhood Cancer Survivor Study)  Country  USA  Data collection  1994 -2007 | Aim:  To characterize the prevalence and risk factors for behaviours consistent with sleep disorders in cancer survivors and examine longitudinal associations with emotional distress and physical health outcomes  Study design:  longitudinal study  Data collection methods: Participants were part of the Childhood Cancer Survivor Study.  Duration followed:  Not reported- minimum 5 years post-diagnosis  Timepoints and intervals  3 timepoints  T0= Baseline (1994)  T1= 6 years post T0 sleep survey (2002)  T3= 11 years post T0 (2007) | Number recruited:  Of 14,355 who completed the baseline survey, 2,645 survivors were randomly selected to complete the sleep survey, and 1933 (73%) completed the sleep survey.  500 siblings were randomly selected to also complete the sleep survey (380 participated; 76%).  Sex:  Male = 47.6%  Diagnosis:  Multiple cancers  Age at diagnosis:  Mean 11.6 years (SD 5.7)  Age at recruitment:  18-29 years n=457, 30-39 years n= 890, 40+ years n=581 | Primary Outcome category:  Functional Health  Domains measured (tool)  Sleep behaviours consistent with sleep disorders (selected items from the PSQI); Sleep management strategies (researcher-created items); daytime sleepiness (ESS); Fatigue (FACIT-F)  Secondary Outcome categories:  Psychological health; Functional health  Domains measured (tool) Emotional distress (Under 18 years: BPI total, 18+ years: BSI-18 depression and anxiety subscales); Physical health conditions (hypertension, headaches, 2^nd^ cancers) (researcher-created items + med record for cancers) | Survivors were more likely to report sleep behaviours consistent with insomnia, sleep-disordered breathing, daytime sleepiness, and frequent use of sleep medications, supplements, and non-behavioral sleep strategies than siblings.  Gender, obesity, age, treatment type, and diagnosis were associated with sleep behaviours and/or sleep management strategies.    Sleep behaviours consistent  with insomnia, sleep-disordered breathing, and daytime sleepiness and fatigue at baseline were related to patterns of late-onset or  persistent psychological distress and survivors reporting these issues were  more likely to develop migraines/ headaches at follow-up. | Barriers:  Biomarkers of cardiovascular functioning could have been used to show more sensitive changes in hypertension  Limitations:  Self‐report may result in recall bias and limit accurately assessing outcome measures | Prospective, repeated analysis of sleep behaviours would provide more information about the nature of survivors’ sleep problems and their role in relation to late effects.  Further study examining specific medications and their duration of  use is necessary to understand how these medications and supplements  impact health and psychosocial functioning. |
| Gibson (2015)  (Childhood Cancer Survivor Study)  Country  USA  Data collection  2003-2014 | Aim:  To examine the smoking status to compare the prevalence of smoking among cancer survivors, siblings, and a comparable general population  Study design: a retrospective cohort study  Data collection methods: Subgroup of the Childhood Cancer Survivor Study who completed a baseline questionnaire  Duration followed:  Mean 12.5 years  Timepoints and interval  3 timepoints  T0=baseline  T1=mean 7.6 years later T2=mean 12.5 years later | Number recruited:  T0 =4997,  T1 =4122,  T2 =3332  (attrition =43%)  Sex:  Male 53.2%  Diagnosis:  multiple cancers  Age at diagnosis:  0-9 years n=4597,  10-12 years n=4800  Age at recruitment:  18-24 years n=3707, 25-34 years n=4470, 35-44 years n=1196, >45 years n=24 | Primary Outcome category:  Functional health  Domains measured (tool)  Self-reported smoking status (2 researcher-created items)  Secondary Outcome categories:  Nil | At baseline smoking rates were lower among survivors (19%) and siblings (24%) compared to the general population (29%). Modest declines in smoking rates were seen at follow-up for all groups.  Characteristics associated with consistent never-smoking included a higher household income, higher education, and cranial radiation therapy.  Psychological distress and heavy alcohol drinking  were inversely associated with consistent never-smoking.  Among ever-smokers, a higher income and education  were associated with quitting, whereas cranial radiation and psychological distress were associated with not having quit.  The development of adverse health conditions was not associated with  smoking patterns. | Barriers:  never-smoking defined as < 100 cigarettes smoked during the lifetime, and this may have misclassified some smokers who had just started smoking  Limitations: the reliance on self-reported smoking status | Effective smoking prevention and cessation interventions are needed for cancer survivors. |
| Gregurek (2009)  Country  Croatia  Data collection  1990-2005 | Aim:  To follow the changes in QoL and anxiety level of patients treated with bone marrow transplantation  Study design: longitudinal design  Data collection methods: Patients were recruited from University hospital  Duration followed:  5 years  Timepoints and intervals  3 timepoints  T0= 3months post-BMT  T1= 12 months post BMT  T3=5 years post BMT | Number recruited:  T0=109,  T1=67,  T2=54,  T3=42  (attrition = 61%)  Sex:  Male 53.2%  Diagnosis:  patients who received a BMT  Age at diagnosis:  Not reported  Age at recruitment: mean 29 years (range 19-53) | Primary Outcome categories:  Functional health; Psychological health  Domains measured (tool)  QoL (KPS); Anxiety (STAI-S & STAI-T)  Secondary Outcome categories:  Education, work, & leisure; Functional health; Intimate relationships, sexuality, & fertility  Domains measured (tool)  Employment status (tool not stated); Body image satisfaction (tool not stated); Sexual functioning satisfaction (tool not stated) | There was a significant increase in QoL over time. It returned to an acceptable level for most, but not all survivors. However, it did not return to their pre-transplant levels.  There was a significant decrease in state and trait anxiety over time.  There was a significant correlation between anxiety and QoL, with lower anxiety associated with higher QoL.  60.7% of patients were employed after BMT but on sick leave, 1 year after BMT 22.4% were employed, and after 5 years 18.6% were employed  Satisfaction with body appearance increased gradually from 70.6% after leaving the hospital to 88.5% after 5 years.  Patients that had BMT were discontented with their sex life, with only 11.8% content after leaving hospital, increasing to 32.7% after 5 years. | Barriers:  All patients during hospitalisation had psychiatric support and they did not have any psychiatric complications during the bone marrow transplantation  Limitations: heterogenic population  Use of self-report questionnaires | Include a psychiatrist in a MDT assisting patients with BMT.  No research recommendations reported |
| Jorngarden (2007)  Part of a research project titled ‘How do adolescents with cancer  cope with disease- and treatment-related aspects of distress?’  Country  Sweden  Data collection  1999-2005 | Aim:  Investigate if and how the HRQoL, anxiety, and depression of a group of adolescents with cancer differ from a reference group at four time-points  Study design: longitudinal design  Data collection methods: Adolescents diagnosed with cancer recruited from 3 paediatric oncology centres. A reference group matched from the general population.  Duration followed:  18 months  Timepoints and interval:  4 time points  T0= shortly after diagnosis  T1=6 months later  T2=12 months later  T3=18 months later | Number recruited:  T0 =56,  T1=53,  T2 =45,  T3 =42  (attrition = 18%)  Sex:  Male 57%  Diagnosis:  Multiple cancers  Age at diagnosis:  13-15 years n=35,  16-21 years n=21  Age at recruitment: not reported | Primary Outcome categories:  Psychological health; Functional health  Domains measured (tool)  Anxiety and depression (HADS anxiety and depression subscales); HRQoL (SF-36 Mental Health and Vitality subscales)  Secondary Outcome categories:  Nil | There is a steady increase in psychological well-being in adolescents diagnosed with cancer from the time of diagnosis.  At baseline, their psychosocial HRQoL and depression were worse than a reference group randomized from the general population.  These differences gradually disappeared and then reversed over 1.5 years after diagnosis.  By the 18-month follow-up, adolescents with cancer had better psychosocial HRQoL, anxiety, and depression than the reference group. | Barriers:  Not reported  Limitations: Small heterogeneous sample size cancer diagnoses, sample excluded patients with recurrence and those who died at some point during data collection, reference group only measured at a single time-point | Because psychosocial adjustment in adolescent cancer survivors changes over time, sensitive longitudinal designs are needed to identify these changes.  Future research is necessary to see if self-reported high psychosocial quality of life and low levels of anxiety and depression at 18 months post-diagnosis persist or whether they wear off as time passes. |
| Lehmann (2014)  (at least 3 previous papers published for this project)  Country  Sweden  Data collection  2003-2013 | Aim:  To provide insight into survivor-reported negative and positive consequences of cancer during adolescence  Study design:  Qualitative longitudinal study  Data collection methods: Participants recruited from 3 university hospitals  Duration followed:  10 years  Timepoints and interval:  3 timepoints  T0= 3 years after diagnosis  T1=4 years after diagnosis  T2= 10 years after diagnosis | Number recruited:  T0=28  T1=25  T2=25  (attrition =11%)  Sex:  Male 54%  Diagnosis:  multiple cancers  Age at diagnosis:  Not reported  Age at recruitment:  mean 25.5 years (range 23-29 years ) | Primary Outcome categories:  Functional health; Psychological health  (These were the main negative and positive consequences hypothesised by the authors a priori)  Domains measured (tool)  Negative and positive consequence of cancer (2 open-ended interview questions)  Secondary Outcome categories:  In the thematic analysis, additional themes arose in the following categories:  Intimate relationships, sexuality, & fertility; Social; Identity & spirituality; Practical & financial issues  Domains measured (tool)  Two open-ended questions (as above) | Most survivors reported both negative and positive consequences at all time points. The categories of these positive and negative consequences changed over time.  Physical problems were the most common negative consequences at all time points.  Additional negative consequences that arose at 10 years included existential concerns, fertility and relationship issues, and concerns relevant to long-term survivorship such as problems with the health care system.  Positive consequences were fairly consistent over time and included positive emotions related to an appreciation of life, a positive sense of self, and closeness with family and friends.  An additional positive consequence at 10 years was joy and gratitude gained from helping others. | Barriers:  Not reported  Limitations:  small sample size, with open-ended questions respondents may forget to mention certain aspects or deliberately chose to avoid talking about certain topics | Future research should address and quantify the consequences of AYA cancer in larger samples and examine whether some consequences are more relevant to specific types of cancer, treatment modalities, gender, or different time points during survival.  Future research should also examine the perceived severity of the reported consequences. |
| Leuteritz (2018)  (AYA-Leipzig study)  Country  Germany  Data collection  2014-2016 | Aim: To describe overall life satisfaction in AYA patients  with cancer by assessing multiple specific life domains  at 2 time points, as well as to detect sociodemographic,  medical, and psychosocial factors associated with life satisfaction in  this group.  Study design: a prospective longitudinal study  Data collection methods: patients recruited from oncological acute care hospitals and rehabilitation clinics  Duration followed:  12 months  Timepoints and interval:  2 timepoints  T0= baseline  T1= 12 months later | Number recruited:  T0 =577,  T1=514  (attrition = 11%)  Sex:  Male 24.9%  Diagnosis:  multiple cancers  Age at diagnosis: 18-25 years n=158,  26-39 years n=356, (mean 29.6 years)  Age at recruitment: not reported | Primary Outcome category:  Functional health  Domains measured (tool)  Life satisfaction (Module A (‘general life satisfaction) of the FLZ-M)  Secondary Outcome categories:  Psychological health; Social  Domains measured (tool)  Perceived adjustment/coping to illness (single item from the PACIS); Illness-specific social support (ISSS-8) | Life satisfaction (LS) was in the upper-middle-range at both time points. It increased significantly over time, but the effect size was small.  About 1/3 of respondents rates each LS domain as unsatisfactory. The lowest LS domains were financial and professional situation, family planning, and sexuality.  Perceived adjustment to illness and illness-specific social support both decreased over time.  Social support was found to be the most decisive factor associated with LS at both time points.  Significant associations with LS were found at baseline for sociodemographic (having children, being in a relationship), medical (diagnosis, comorbidities, sick leave, time since diagnosis, treatment status), and psychosocial variables (social support, adjustment to illness). | Barriers:  Not reported  Limitations: majority-female; heterogeneous sample with regard to diagnosis, treatment, and time since diagnosis; some relevant medical information not collected (e.g. tumour stage); separating the domains of the FLZ-M may have impacted its psychometric properties; refusal rate was not systematically documented at all recruitment settings | Future research: expand the current study to include additional parameters such as coping and self-efficacy and empowerment in AYAs. |

HRQoL = Health-related quality of life

MOS= Medical Outcomes Study approach

GSI-BSI-18= Global Symptom Index of the Brief Symptom Inventory-18

SF-36 = Short Form 36 Physical and Mental Health Summary Scales

TACQOL = TNO (Netherlands Organization for Applied Scientific Research) and AZL (Leiden University Medical Center) Children’s Quality of Life Questionnaire

TAAQOL = TNO and AZL Adult’s Quality of Life Questionnaire

BT-DUX = Bone tumor version of the DUX-25 (short form of the DUCATQOL=Dutch Children AZL/TNO Questionnaire Quality Of Life)

TESS = Toronto Extremity Salvage Score

TUDS = Timed up and down the stairs

VWA = Combination of three various walking activities (10m normal, slalom, obstacle walking)

6 MWT = 6-minute walk test

PCI = Physiological cost index

ActiLog = Activity monitor

GPA = General Physical Activity Score

CCSS-NCQ = Childhood Cancer Survivor Study Neurocognitive Questionnaire

BSI-18 = Brief Symptom Inventory-18

PDS = Post-traumatic Stress Diagnostic Scale

WAI = Work Ability Index

COPSOQ = Copenhagen Psychosocial Questionnaire

IOC = Impact of Cancer instrument

PSQI = Pittsburgh Sleep Quality Index

ESS = Epworth Sleepiness Scale

FACIT-F = Functional Assessment of Chronic Illness Therapy‐Fatigue

KPS = Karnofsky Performance Scale

STAI-S = Spielberger State-Trait Anxiety Inventory – State Subscale

STAI-T = Spielberger State-Trait Anxiety Inventory – Trait Subscale

HADS = Hospital Anxiety and Depression Scale

FLZ-M = Questions on Life Satisfaction

PACIS = Perceived Adjustment to Chronic Illness Scale

ISSS-8 = Illness-Specific Social Support Scale Short Version-8

## References

Acquati, C., Zebrack, B. J., Faul, A. C., Embry, L., Aguilar, C., Block, R., Hayes-Lattin, B., Freyer, D. R., & Cole, S. (2018). Sexual Functioning Among Young Adult Cancer Patients: A 2-Year Longitudinal Study. *Cancer, 124*(2), 398-405. <https://doi.org/10.1002/cncr.31030>

Armuand, G., Wettergren, L., Nilsson, J., Rodriguez-Wallberg, K., & Lampic, C. (2018). Threatened fertility: A longitudinal study exploring experiences of fertility and having children after cancer treatment. *European Journal of Cancer Care, 27*(2). <https://doi.org/10.1111/ecc.12798>

Bekkering, W. P., Vlieland, T., Koopman, H. M., Schaap, G. R., Beishuizen, A., Anninga, J. K., Wolterbeek, R., Nelissen, R., & Taminiau, A. H. M. (2012). A prospective study on quality of life and functional outcome in children and adolescents after malignant bone tumor surgery. *Pediatric blood & cancer, 58*(6), 978-985. <https://doi.org/10.1002/pbc.23328>

Brinkman, T. M., Lown, E. A., Li, C. H., Olsson, I. T., Marchak, J. G., Stuber, M. L., Vuotto, S., Srivastava, D., Nathan, P. C., Leisenring, W. M., Armstrong, G. T., Robison, L. L., & Krull, K. R. (2019). Alcohol consumption behaviors and neurocognitive dysfunction and emotional distress in adult survivors of childhood cancer: a report from the Childhood Cancer Survivor Study. *Addiction, 114*(2), 226-235. <https://doi.org/10.1111/add.14439>

Brock, H., Friedrich, M., Sender, A., Richter, D., Geue, K., Mehnert-Theuerkauf, A., & Leuteritz, K. (2021, Jun 12). Work ability and cognitive impairments in young adult cancer patients: associated factors and changes over time-results from the AYA-Leipzig study. *J Cancer Surviv*. <https://doi.org/10.1007/s11764-021-01071-1>

Cappelli, C., Miller, K. A., Ritt-Olson, A., Pentz, M. A., Salahpour, S., & Milam, J. E. (2021, Sep-Oct). Binge Drinking, Tobacco, and Marijuana Use Among Young Adult Childhood Cancer Survivors: A Longitudinal Study. *J Pediatr Oncol Nurs, 38*(5), 285-294. <https://doi.org/10.1177/10434542211011036>

Cho, D., & Park, C. L. (2015). Cancer-related identities in people diagnosed during late adolescence and young adulthood. *British Journal of Health Psychology, 20*(3), 594-612. <https://doi.org/10.1111/bjhp.12110>

Daniel, L. C., Wang, M. J., Mulrooney, D. A., Srivastava, D. K., Schwartz, L. A., Edelstein, K., Brinkman, T. M., Zhou, E. S., Howell, R. M., Gibson, T. M., Leisenring, W., Oeffinger, K. C., Neglia, J., Robison, L. L., Armstrong, G. T., & Krull, K. R. (2019). Sleep, emotional distress, and physical health in survivors of childhood cancer: A report from the Childhood Cancer Survivor Study. *Psycho-oncology, 28*(4), 903-912. <https://doi.org/10.1002/pon.5040>

Gibson, T. M., Liu, W., Armstrong, G. T., Srivastava, D. K., Hudson, M. M., Leisenring, W. M., Mertens, A. C., Klesges, R. C., Oeffinger, K. C., Nathan, P. C., & Robison, L. L. (2015). Longitudinal smoking patterns in survivors of childhood cancer: An update from the Childhood Cancer Survivor Study. *Cancer, 121*(22), 4035-4043. <https://doi.org/10.1002/cncr.29609>

Gregurek, R., Brajkovic, L., Kalenic, B., Bras, M., & Persic-Brida, M. (2009). Five years study on impact of anxiety on quality of life in patients treated with bone marrow transplantation. *Psychiatria Danubina, 21*(1), 49-55. <https://ezproxy.library.usyd.edu.au/login?url=http://ovidsp.ovid.com/ovidweb.cgi?T=JS&CSC=Y&NEWS=N&PAGE=fulltext&D=psyc6&AN=2009-05126-007>

Jorngarden, A., Mattsson, E., & von Essen, L. (2007). Health-related quality of life, anxiety and depression among adolescents and young adults with cancer: A prospective longitudinal study. *European Journal of Cancer, 43*(13), 1952-1958. <https://doi.org/10.1016/j.ejca.2007.05.031>

Lehmann, V., Gronqvist, H., Engvall, G., Ander, M., Tuinman, M. A., Hagedoorn, M., Sanderman, R., Mattsson, E., & von Essen, L. (2014). Negative and positive consequences of adolescent cancer 10 years after diagnosis: an interview-based longitudinal study in Sweden. *Psycho-oncology, 23*(11), 1229-1235. <https://doi.org/10.1002/pon.3549>

Leuteritz, K., Friedrich, M., Sender, A., Nowe, E., Stoebel-Richter, Y., & Geue, K. (2018). Life satisfaction in young adults with cancer and the role of sociodemographic, medical, and psychosocial factors: Results of a longitudinal study. *Cancer, 124*(22), 4374-4382. <https://doi.org/10.1002/cncr.31659>
