# Supplementary material for: Psychological, functional and social outcomes in adolescent and young adult cancer survivors over time: A systematic review of longitudinal studies
Source: Psychooncology. 2022 Jul 2;31(9):1448–58. doi: 10.1002/pon.5987 (PMC9544373; doi:10.1002/pon.5987)
Supplement: Supplementary file 3 — Supporting Information S3 [file PON-31-1448-s002.docx]

| **QATDSD Criteria** | **Number of studies scored (%) ^references^** | | | | |
| --- | --- | --- | --- | --- | --- |
|  | **Not at all** | **Very slightly**  **1** | **Moderately**  **2** | **Completely**  **3** | **Not applicable to article type** |
| 1. Explicit theoretical framework | 1 (8%) ^Bekkering et al., 2012^ | 5 (38%) ^Brinkman et al., 2019; Cappelli et al., 2021; Daniel et al., 2019; Gibson et al., 2015; Gregurek et al., 2009^ | 3 (23%) ^Acquati et al., 2018; Brock et al., 2021; Jorngarden et al., 2007^ | 4 (23%) ^Armuand et al., 2018; Cho & Park, 2015; Lehmann et al., 2014; Leuteritz et al., 2018,^ | - |
| 1. Statement of aims/objectives | - | - | 7 (54%) ^Acquati 2018, Bekkering 2012, Brock 2021, Daniel 2019, Gregurek 2009, Jorngarden 2007, Lehmann 2014^ | 6 (46%) ^Armuand 2018, Brinkman 2019, Capelli 2021, Cho 2015, Gibson 2015, Leuteritz 2018^ | - |
| 1. Clear description of research setting | 1 (8%) ^Gibson 2015^ | 2 (15%) ^Cho 2015, Daniel 2019^ | 6 (46%) ^Acquati 2018, Brinkman 2019, Brock 2021, Gregurek 2009, Lehmann 2014, Leuteritz 2018^ | 4 (31%) ^Armuand 2018, Bekkering 2012, Capelli 2021, Jorngarden 2007^ | - |
| 1. Evidence of sample size considered in terms of analysis | 4 (31%) ^Acquati 2018, Brock 2021, Gregurek 2009, Leuteritz 2018^ | 5 (38%) ^Brinkman 2019, Capelli 2021, Cho 2015, Jorngarden 2007, Lehmann 2014^ | 2 (15%) ^Bekkering 2012, Daniel 2019^ | 2 (15%) ^Armuand 2018, Gibson 2015^ | - |
| 1. Representative sample of target group of a reasonable size | - | 5 (38%) ^Bekkering 2012, Cho 2015, Gregurek 2009, Jorngarden 2007, Lehmann 2014^ | 6 (46%) ^Acquati 2018, Armuand 2018, Brock 2021, Capelli 2021, Daniel 2019, Leuteritz 2018^ | 2 (15%) ^Brinkman 2019, Gibson 2015^ | - |
| 1. Description of procedure for data collection | 1 (8%) ^Daniel 2019^ | 4 (31%) ^Acquati 2018, Brinkman 2019, Capelli 2021, Gibson 2015^ | 1 (8%) ^Gregurek 2009^ | 7 (54%) ^Armuand 2018, Bekkering 2012, Brock 2021, Cho 2015, Jorngarden 2007, Lehmann 2014, Leuteritz 2018^ | - |
| 1. Rationale for choice of data collection tool(s) | 1 (8%) ^Gibson 2015^ | 1 (8%) ^Lehmann 2014^ | 4 (31%) ^Cho 2015, Daniel 2019, Gregurek 2009, Leuteritz 2018^ | 7 (54%) ^Acquati 2018, Armuand 2018, Bekkering 2012, Brinkman 2019, Brock 2021, Capelli 2021, Jorngarden 2007^ | - |
| 1. Detailed recruitment data | - | 6 (46%) ^Acquati 2018, Capelli 2021, Daniel 2019, Gibson 2015, Gregurek 2009, Lehmann 2014^ | 4 (31%) ^Brinkman 2019, Brock 2021, Cho 2015, Leuteritz 2018^ | 3 (23%) ^Armuand 2018, Bekkering 2012, Jorngarden 2007^ | - |
| 1. Statistical assessment of reliability and validity of measurement tool(s) (Quantitative) | 3 (23%) ^Daniel 2019, Gibson 2015, Gregurek 2009^ | - | 6 (46%) ^Acquati 2018, Bekkering 2012, Brinkman 2019, Brock 2021, Capelli 2021, Jorngarden 2007^ | 2 (15%) ^Cho 2015, Leuteritz 2018^ | 2 (15%) ^Armuand 2018, Lehmann 2014^ |
| 1. Fit between stated research question and method of data collection (Quantitative) | - | - | 9 (69%) ^Acquati 2018, Brinkman 2019, Brock 2021, Capelli 2021, Cho 2015, Daniel 2019, Gregurek 2009, Jorngarden 2007, Leuteritz 2018^ | 2 (15%) ^Bekkering 2012, Gibson 2015^ | 2 (15%) ^Armuand 2018, Lehmann 2014^ |
| 1. Fit between stated research question and content of data collection tool (Qualitative) | - | 1 (8%) ^Lehmann 2014^ | 1 (8%) ^Armuand 2018^ | - | 11 (85%) ^Acquati 2018, Bekkering 2012, Brinkman 2019, Brock 2021, Capelli 2021, Cho 2015, Daniel 2019, Gibson 2015, Gregurek 2009, Jorngarden 2007, Leuteritz 2018^ |
| 1. Fit between research question and method of analysis | - | 3 (23%) ^Brock 2021, Cho 2015, Gregurek 2009^ | 6 (46%) ^Bekkering 2012, Brinkman 2019, Daniel 2019, Jorngarden 2007, Lehmann 2014, Leuteritz 2018^ | 4 (31%) ^Acquati 2018, Armuand 2018, Capelli 2021, Gibson 2015^ | - |
| 1. Good justification for analytical method selected | - | 3 (23%) ^Gregurek 2009, Jorngarden 2007, Lehmann 2014^ | 7 (54%) ^Bekkering 2012, Brock 2021, Capelli 2021, Cho 2015, Daniel 2019, Gibson 2015, Leuteritz 2018^ | 3 (23%) ^Acquati 2018, Armuand 2018, Brinkman 2019^ | - |
| 1. Assessment of reliability of analytical process (Qualitative) | 1 (8%) ^Lehmann 2014^ | - | - | 1 (8%) ^Armuand 2018^ | 11 (85%) ^Acquati 2018, Bekkering 2012, Brinkman 2019, Brock 2021, Capelli 2021, Cho 2015, Daniel 2019, Gibson 2015, Gregurek 2009, Jorngarden 2007, Leuteritz 2018^ |
| 1. Evidence of user involvement in design | 13 (100%) ^Acquati 2018, Armuand 2018, Bekkering 2012, Brinkman 2019, Brock 2021, Capelli 2021, Cho 2015, Daniel 2019, Gibson 2015, Gregurek 2009, Jorngarden 2007, Lehmann 2014, Leuteritz 2018^ | - | - | - | - |
| 1. Strengths and limitations critically discussed | - | 2 (15%) ^Gregurek 2009, Lehmann 2014^ | 8 (62%) ^Acquati 2018, Armuand 2018, Brinkman 2019, Brock 2021, Capelli 2021, Daniel 2019, Jorngarden 2007, Leuteritz 2018^ | 3 (23%) ^Bekkering 2012, Cho 2015, Gibson 2015^ | - |

## References

Acquati, C., Zebrack, B. J., Faul, A. C., Embry, L., Aguilar, C., Block, R., Hayes-Lattin, B., Freyer, D. R., & Cole, S. (2018). Sexual Functioning Among Young Adult Cancer Patients: A 2-Year Longitudinal Study. *Cancer, 124*(2), 398-405. <https://doi.org/10.1002/cncr.31030>

Armuand, G., Wettergren, L., Nilsson, J., Rodriguez-Wallberg, K., & Lampic, C. (2018). Threatened fertility: A longitudinal study exploring experiences of fertility and having children after cancer treatment. *European Journal of Cancer Care, 27*(2). <https://doi.org/10.1111/ecc.12798>

Bekkering, W. P., Vlieland, T., Koopman, H. M., Schaap, G. R., Beishuizen, A., Anninga, J. K., Wolterbeek, R., Nelissen, R., & Taminiau, A. H. M. (2012). A prospective study on quality of life and functional outcome in children and adolescents after malignant bone tumor surgery. *Pediatric blood & cancer, 58*(6), 978-985. <https://doi.org/10.1002/pbc.23328>

Brinkman, T. M., Lown, E. A., Li, C. H., Olsson, I. T., Marchak, J. G., Stuber, M. L., Vuotto, S., Srivastava, D., Nathan, P. C., Leisenring, W. M., Armstrong, G. T., Robison, L. L., & Krull, K. R. (2019). Alcohol consumption behaviors and neurocognitive dysfunction and emotional distress in adult survivors of childhood cancer: a report from the Childhood Cancer Survivor Study. *Addiction, 114*(2), 226-235. <https://doi.org/10.1111/add.14439>

Brock, H., Friedrich, M., Sender, A., Richter, D., Geue, K., Mehnert-Theuerkauf, A., & Leuteritz, K. (2021, Jun 12). Work ability and cognitive impairments in young adult cancer patients: associated factors and changes over time-results from the AYA-Leipzig study. *J Cancer Surviv*. <https://doi.org/10.1007/s11764-021-01071-1>

Cappelli, C., Miller, K. A., Ritt-Olson, A., Pentz, M. A., Salahpour, S., & Milam, J. E. (2021, Sep-Oct). Binge Drinking, Tobacco, and Marijuana Use Among Young Adult Childhood Cancer Survivors: A Longitudinal Study. *J Pediatr Oncol Nurs, 38*(5), 285-294. <https://doi.org/10.1177/10434542211011036>

Cho, D., & Park, C. L. (2015). Cancer-related identities in people diagnosed during late adolescence and young adulthood. *British Journal of Health Psychology, 20*(3), 594-612. <https://doi.org/10.1111/bjhp.12110>

Daniel, L. C., Wang, M. J., Mulrooney, D. A., Srivastava, D. K., Schwartz, L. A., Edelstein, K., Brinkman, T. M., Zhou, E. S., Howell, R. M., Gibson, T. M., Leisenring, W., Oeffinger, K. C., Neglia, J., Robison, L. L., Armstrong, G. T., & Krull, K. R. (2019). Sleep, emotional distress, and physical health in survivors of childhood cancer: A report from the Childhood Cancer Survivor Study. *Psycho-oncology, 28*(4), 903-912. <https://doi.org/10.1002/pon.5040>

Gibson, T. M., Liu, W., Armstrong, G. T., Srivastava, D. K., Hudson, M. M., Leisenring, W. M., Mertens, A. C., Klesges, R. C., Oeffinger, K. C., Nathan, P. C., & Robison, L. L. (2015). Longitudinal smoking patterns in survivors of childhood cancer: An update from the Childhood Cancer Survivor Study. *Cancer, 121*(22), 4035-4043. <https://doi.org/10.1002/cncr.29609>

Gregurek, R., Brajkovic, L., Kalenic, B., Bras, M., & Persic-Brida, M. (2009). Five years study on impact of anxiety on quality of life in patients treated with bone marrow transplantation. *Psychiatria Danubina, 21*(1), 49-55. <https://ezproxy.library.usyd.edu.au/login?url=http://ovidsp.ovid.com/ovidweb.cgi?T=JS&CSC=Y&NEWS=N&PAGE=fulltext&D=psyc6&AN=2009-05126-007>

Jorngarden, A., Mattsson, E., & von Essen, L. (2007). Health-related quality of life, anxiety and depression among adolescents and young adults with cancer: A prospective longitudinal study. *European Journal of Cancer, 43*(13), 1952-1958. <https://doi.org/10.1016/j.ejca.2007.05.031>

Lehmann, V., Gronqvist, H., Engvall, G., Ander, M., Tuinman, M. A., Hagedoorn, M., Sanderman, R., Mattsson, E., & von Essen, L. (2014). Negative and positive consequences of adolescent cancer 10 years after diagnosis: an interview-based longitudinal study in Sweden. *Psycho-oncology, 23*(11), 1229-1235. <https://doi.org/10.1002/pon.3549>

Leuteritz, K., Friedrich, M., Sender, A., Nowe, E., Stoebel-Richter, Y., & Geue, K. (2018). Life satisfaction in young adults with cancer and the role of sociodemographic, medical, and psychosocial factors: Results of a longitudinal study. *Cancer, 124*(22), 4374-4382. <https://doi.org/10.1002/cncr.31659>
